# Feasibility, Affordability, Benefits, and Challenges of Transitioning to Virtual Case Management for PLHIV: Experiences From Indonesia and Nepal

**DOI:** 10.9745/GHSP-D-24-00027

**Published:** 2026-08-10

**Authors:** Elizabeth Costenbader, Shanthi Noriega Minichiello, Matthew Zinck, Kiran Bam, Bhagawan Shrestha, Caroline Francis, Benjamin Eveslage, Rick Homan

**Affiliations:** a FHI 360, Washington, DC, USA. Now with Costenbader Collaborative Consulting, Washington, DC, USA.; b FHI 360, Washington, DC, USA. Now with University of California, Berkeley, Berkeley, CA, USA.; c FHI 360, Durham, NC, USA. Now with Gillings School of Global Public Health, University of North Carolina, Chapel Hill, NC, USA.; d FHI 360, Kathmandu, Nepal. Now with La Trobe University, Melbourne, Australia.; e FHI 360 Nepal, Kathmandu, Nepal.; f FHI 360 Indonesia, Jakarta, Indonesia.; g FHI 360, Durham, NC, USA.; h FHI 360, Durham, NC, USA. Now retired.

## Abstract

**Background:**

HIV case management supports people living with HIV to be linked to and retained in care. HIV case management becomes virtual case management (VCM) when some or all these services are provided via virtual tools or platforms including mobile phones, chat messenger apps, other mobile or web apps, and/or electronic case management systems. The COVID-19 pandemic accelerated the use and expansion of virtual services, including health services, across the globe.

**Program Description:**

These case studies document how HIV programs in Indonesia and Nepal transitioned from in-person case management to VCM during the COVID-19 pandemic in 2020. As part of this transition, the program in Nepal implemented VCM in 37 districts that were providing services to 12,820 people living with HIV (PLHIV), and in Indonesia the program supported 18,244 PLHIV in 5 districts. Data on the transition from in-person support to VCM were collected on client clinical outcomes, costs of the virtual services, and case manager feedback.

**Results:**

These case studies indicate that VCM is a feasible and affordable approach to implement in low- and middle-income country contexts. Between March and September 2020, 10,995 (82.1%) and 3,801 (51.5%) PLHIV in Nepal and Indonesia, respectively, were supported by VCM. Continuity of care remained high in both countries with only 1%–2% interruptions in treatment. Viral load suppression also remained high in both countries (91%–92% in Indonesia and 94%–95% in Nepal). Viral load coverage, however, was low, but this was influenced by clinic and laboratory closures. Startup costs were low (<US$2,500), and the monthly cost per person to provide VCM was $2.34 in Nepal and $4.67 in Indonesia. Case manager feedback was focused on compensation, expanded reach of virtual services, gaps in client mobile phone ownership or digital literacy, efficiency of services, and confidentiality and privacy.

**Conclusion:**

VCM has transformative potential to expand the reach of health services and provide differentiated options, meeting clients’ preferences and needs. The experiences and lessons learned from these two case studies can guide the implementation of future VCM programs in other countries.

## Abstract (precis)

Virtual case management was an effective, feasible, and affordable approach to support HIV care services in Indonesia and Nepal during the COVID-19 pandemic. Experiences and lessons from these case studies can help other country programs implement virtual case management services.

## Teaser

**Key Findings:**

- Virtual case management was an effective approach to maintaining clinical outcomes for people living with HIV (PLHIV), with only 1%–2% interruptions in treatment and 91%–95% viral load suppression in Indonesia and Nepal.
- Virtual case management was affordable with low startup costs (<US$2500) and low monthly costs per PLHIV supported (US$2.34 to $4.67 per client).

**Key Implications:**

- Virtual case management is an effective and financially viable approach for low- and middle-income countries to expand HIV case management service reach.
- Workforce compensation structures may need to be adapted for successful implementation of virtual case management, as case managers typically receive travel-related compensation for in-person services that may not apply to virtual service delivery.
- Some clients may not have been able to access virtual services because they lacked internet connectivity, mobile phones, and/or digital literacy.
- A hybrid model combining virtual and in-person case management has the potential to expand the reach and efficiency of HIV care services while providing differentiated options that meet clients’ needs and preferences.

## Background

Advances in antiretroviral therapy (ART) over the past 25 years have made it possible for people living with HIV (PLHIV) to live a near-normal life expectancy.^1^^,^^2^ PLHIV must be consistently engaged in care and able to access uninterrupted ART to achieve and maintain viral suppression throughout their lives.

Virtual communication channels have become increasingly commonplace over the past decade, changing the landscape of how people connect to and access information and services. In health care settings, COVID-19-related lockdowns and social distancing requirements meant virtual channels rapidly became necessary and, often, the predominant means of providing care.^3^

FHI 360 and partners worked to develop a model for introducing virtual approaches to HIV-related services across the HIV treatment cascade from initial diagnosis to achieving viral suppression.^4^^,^^5^ This model was subsequently adapted and published in guidance from global normative bodies.^6^ FHI 360’s updated telehealth framework, which spans the client care continuum and has been adapted to be relevant beyond HIV, illustrates how virtual approaches can support clients from initial planning and outreach through service delivery, re-engagement, and quality improvement (Figure).

**FIGURE:**
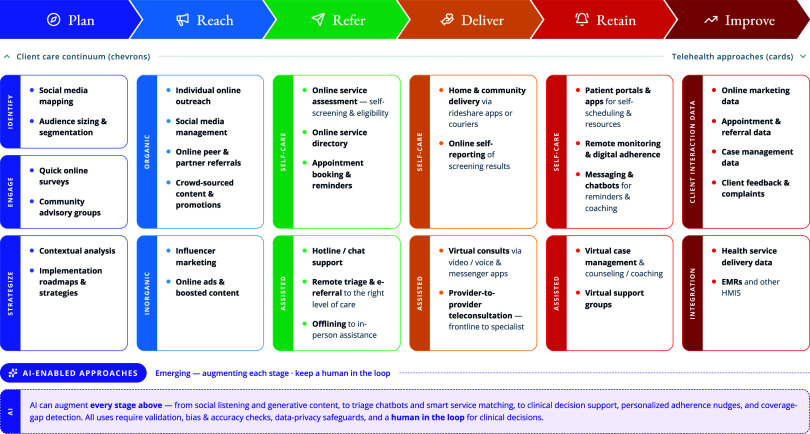
FHI 360 Framework: Telehealth Approaches Across the Client Care Continuum Abbreviations: AI, artificial intelligence; EMR, electronic medical record; HMIS, health management information system. Six sequential stages of the client care continuum (chevrons: Plan, Reach, Refer, Deliver, Retain, Improve) are mapped to the telehealth approaches that support clients at each stage (cards). Approaches are grouped as *self-care* (client-led) or *assisted* (involving a provider or health worker); for the Reach stage, *organic* denotes unpaid audience-led growth and *inorganic* denotes paid promotion. A cross-cutting layer of emerging *AI-enabled* approaches can augment every stage. Cross-cutting enablers apply throughout, including informed consent and data protection and attention to the digital divide (literacy, language, and device and connectivity access).

HIV case management is a relationship between a case manager and client in their cohort. The case manager provides tailored support to clients in their cohort to track progress and achieve specific clinical goals.^7^ The Figure shows the role of case management across the HIV services cascade. The role of case management may differ across settings, but the overarching goal is to provide individualized assistance to clients to improve their health. Case management is critical to improved outcomes, and several studies in different settings have demonstrated that case management improves care engagement and clinical outcomes and is acceptable to both clients and clinical teams.^6^^, ^^8^^–^^12^

Case management becomes virtual case management (VCM) when case managers provide some or all support to clients through virtual platforms such as mobile phones, messaging apps, and online reservation systems/electronic case management systems.^6^ VCM can be used during the refer, deliver, re-engage, and improve stages of the cascade, such as phone- or video-based post-test counseling and ART and PrEP-related counseling. Also, electronic case management systems can allow clients and case managers to book services online.^6^

To date, little has been published on VCM in low- and middle-income country (LMIC) contexts. Comprehensive documentation is necessary to better inform feasibility, acceptability, cost, and sustainability of this approach and to establish best practices. This article documents the experience of HIV programs in two countries, Indonesia and Nepal, as they converted HIV case management from in-person to virtual during the COVID-19 pandemic.

## Relevant Context in Indonesia and Nepal

### Epidemic Context

In 2020, the HIV prevalence in Nepal and Indonesia was less than 1%, with 30,300 and 543,100 PLHIV in Nepal and Indonesia, respectively.^13^^,^^14^ In both countries, the HIV epidemics were concentrated, with prevalence substantially higher among certain populations than in the general population. HIV services in both countries were accordingly designed for populations disproportionately affected by HIV, in accordance with national epidemiology and program priorities at the time.

### Policy and Digital Context

Both countries had existing national policies and guidelines that informed their HIV response before and during the COVID pandemic, including the support of differentiated service delivery. In Nepal, the National Centre for AIDS and STD Control (NCASC) supported investments in an HIV Care and ART Tracking System (i.e., DHIS2 Tracker), mHealth initiatives, and a biometric system linked to DHIS2. The NCASC also recommended multi-month dispensing of antiretrovirals for clinically well PLHIV.^13^ The NCASC also released guidelines for an HIV Standard Service Package for Key Populations in 2020. These guidelines emphasized differentiated service delivery including community and home-based care via community health workers providing linkage to care, care navigation, adherence and ART retention support, transportation and accompaniment, psychosocial support, referral, and other follow-up services.^15^ The standard service package also endorsed community-based ART distribution sites and “e-Reach,” which included the use of various online platforms to expand reach to previously unreached individuals.^15^ The Nepal Ministry of Health also had the National e-Health Strategy 2017, which provided a mission and vision, along with key implementation priorities, for e-Health in Nepal.^16^ The strategy focused on institutionalizing and strengthening telehealth services, using technology to improve care for clients, creating an enabling environment for e-Health, and increasing the capacity of health workers and systems to implement e-Health.^16^

In Indonesia, due to the onset of the COVID-19 pandemic, the Provincial Health Office in Jakarta released a sub-national emergency circular, which transitioned community-based HIV services from face-to-face contacts to virtual contacts. It also endorsed two-month antiretroviral dispensing and home-based antiretroviral delivery services.^17^ Additional comparable information about policies or guidelines that were developed in response to the COVID-19 pandemic and could have influenced VCM delivery in Indonesia were unobtainable.

The existing digital landscape is also important context for implementing VCM programs. In 2020, only 35% and 64% of the population in Nepal and Indonesia, respectively, had access to the internet.^18^^,^^19^ However, both countries had high mobile phone use in 2020, with 148% and 124% mobile subscriptions per population in Nepal and Indonesia, respectively.^18^^,^^19^ These subscription rates of over 100% indicate that in both countries some people had more than one mobile device. However, the distribution of mobile phone ownership and access is unclear from this data.

## Program Adaptation and Rollout

Prior to the pandemic, in both Nepal and Indonesia, HIV case management was provided primarily in person by trained community-based staff, who worked in collaboration with facility-based case managers to reach and support clients. Case managers coordinated with the community-based staff to identify and assign necessary follow-up for clients in between facility visits.

The process of transitioning to VCM for both country programs was guided by the Going Online Vision, a report developed under the LINKAGES and EpiC projects and published in 2019 by FHI 360.^20^ In Indonesia, the LINKAGES project was funded by the U.S. Government and delivered by FHI 360 from 2015 through 2021. LINKAGES focused on providing HIV prevention and treatment programs among populations at increased risk of HIV. The subsequent Meeting Targets and Maintaining Epidemic Control (EpiC) project (2019–2025) built on the foundation laid by the LINKAGES project, in Nepal and elsewhere, as a global initiative focused on achieving HIV epidemic control and self-reliant management of national HIV programs by improving HIV case finding, prevention, and treatment programming. The Going Online Vision, developed under these projects, defined a framework for planning, implementing, and monitoring HIV services delivered through virtual channels, which guided the VCM program development and adaptation in both locations.^20^

In 2020, EpiC Nepal, which was then providing technical support to 37 districts that provided services to 12,820 PLHIV, implemented VCM in all supported districts.^21^ In 2020, LINKAGES Indonesia implemented VCM in 5 districts in Jakarta: North Jakarta, South Jakarta, East Jakarta, West Jakarta, and Central Jakarta. At that time, LINKAGES Indonesia was supporting 18,244 PLHIV across 60 PEPFAR-supported facilities in these 5 districts.^22^

### Nepal Transition

Global normative bodies declared COVID-19 a pandemic on March 11, 2020.^23^ Following this declaration, EpiC Nepal expanded their existing VCM services, with 83 of 113 employed case managers transitioning from providing primarily in-person support to PLHIV to VCM support over a period of several weeks based on local restrictions and client preferences. Clients had the option to transition to VCM or remain with modified in-person services per local restrictions and/or approval from local authorities. The Nepal team disseminated VCM guidance to community partners, began holding virtual support groups and adherence club meetings, integrated telephone follow-up for PLHIV, coordinated community/home distribution of ART, and adapted existing tracking tools. Specifically, they adapted standard operating procedures (SOPs) and digitized forms that were used to track case managers’ individual interactions with PLHIV. Also, case managers adapted a tracker tool used by online outreach workers to include case management-related indicators. Case managers were given refresher trainings on case management services as well as online outreach and motivational counseling to effectively engage clients virtually. In Nepal, the transition from in-person support to VCM was facilitated by the program’s earlier experience in online outreach and services, namely the development and use of MeroSathi, a web-based online reservation and case management application allowing clients to book services.

Existing clients who elected were transitioned to VCM services. New clients were recruited through various strategies including limited community-based organization engagement, community outreach programs, and online marketing and outreach, with more emphasis on the latter over time (Table 1). The goal of the first two strategies was to link clients from offline (community-based) to online services. Although community-based activities were limited, outreach was conducted by community-based supporters and peer navigators, and at community events and adherence clubs. As the pandemic progressed, efforts to shift to online outreach were strengthened. Online marketing efforts focused on social media platforms (e.g., Facebook), messenger apps, and dating apps. Marketing content and messaging were focused on HIV awareness, benefits of ART and viral load suppression, and providing links to MeroSathi. Social media influencers and virtual champions were mobilized to reinforce the online marketing messaging, expand reach and engagement, and build trust among online populations at increased risk of HIV. Case managers contacted clients through messenger apps (e.g., WhatsApp) or via cellular mobile networks for their VCM sessions.

**TABLE 1.** Approaches to Client Recruitment, Identification and Outreach, and Contact for In-Person vs. Virtual HIV Case Management in Nepal and Indonesia

**Country**	**Mode of Service Provision**	**Recruitment Strategies**	**Identification and Outreach**	**Contact**
Nepal	In-Person	Community outreach programs	Case managers coordinated with peer navigators and physically identified and followed up with clients	Face-to-face meetings in community or at facilities
	Virtual	Transition of existing clients Online marketing and outreach Community outreach programs Community-based organization engagement	Peer navigators and influencers identified and refer potential clients for VCM Used virtual hotspot mapping and online outreach tracking tools	Developed job aids including educational materials/videos and pre-scripted, tailored messages Used WhatsApp and other messenger apps, telephone calls, and SMS
Indonesia	In-Person	Referral of newly diagnosed clients	Facility staff and/or outreach workers initiated identification and referral	Face-to-face meetings at facilities
	Virtual	Transition of existing clients Referral of newly diagnosed clients during period Online marketing and outreach	Used case management intake form to assess length of prior ART treatment and identify any custom support needed	Used WhatsApp (voice and text), telephone calls, and SMS, and other messenger apps, as per client preferences

Abbreviations: ART, antiretroviral therapy; VCM, virtual case management.

### Indonesia Transition

The first COVID-19 case was officially declared in Indonesia on March 2, 2020.^24^ As the first wave of lockdowns in Jakarta were put into place, face-to-face case management with PLHIV became increasingly untenable. The LINKAGES Indonesia project and its 6 community implementers spent one month making the transition to VCM. Most case management services were subsequently offered through VCM per the Jakarta Provincial Health Office circular, or subnational policy (No. 57 / SE / 2020), which placed restrictions on face-to-face services, but there were some situations where in-person support was deemed necessary. LINKAGES Indonesia quickly established technical guidance and SOPs for delivering VCM services for PLHIV at different stages of ART and used Google Forms to transfer case management data from 53 case managers to their respective community service organization (CSO) monitoring & evaluation personnel. These personnel were trained on use of SOPs and Google Forms, and subsequently, the CSOs took the lead in providing the VCM services to their clients.

The case managers documented their activities using the Google Forms and the CSOs continued to supervise, monitor performance, and coordinate activities as the pandemic unfolded and services changed in response. The FHI 360 and CSO teams held meetings every 2 weeks to ensure that any problems could be identified and addressed for uninterrupted service access. Training and mentoring support were provided virtually each month, and online coordination meetings held every 2 weeks provided opportunities to further adapt client-centered programming within the dynamic COVID-19 context.

Existing case management clients were transitioned from face-to-face support to VCM. Newly diagnosed PLHIV were referred to VCM services through facility staff and/or outreach workers (Table 1). Case managers discussed client communication and support preferences during VCM transition (existing clients) or orientation (new clients) sessions due to the large-scale social restrictions in place in the country. Online community-based supporters also conducted online outreach and provided information on how to contact case managers and be connected to VCM services. Case managers used WhatsApp (voice and text), telephone, SMS, and other messenger apps to communicate with clients, based on client preferences.

#### VCM Services

As shown in Table 2, the 4 main HIV case management services provided to clients before the pandemic in both Nepal and Indonesia were support for ART adherence, psychosocial and mental health support, coordination of ART refills, and connecting clients, by accompaniment and/or informational support, to viral load (VL) testing and other medical services. Prior to the rollout of VCM, these case management services were typically provided during appointments at the clinic or home visits. In both countries, all these services were transitioned to phone or internet delivery platforms and continued to be offered under VCM.

**TABLE 2.** Distinctions Between Online and Virtual HIV Case Management in Nepal and Indonesia

**Country**	**Mode of Service Provision**	**HIV Case Management Services**			
		**Support for ART Adherence**	**Psychosocial and Mental Health Support**	**Coordination of ART refills**	**Link to Viral Load Testing**
Nepal	In-person	Visits to clients’ homes	In-person support through quarterly individual meetings with clients and support group sessions held at the community organization	In-person orders followed by home delivery or clinic visit to pick up refill	Sign-up at clinic
	Virtual	Voice calls or online messenger chats, followed by assistance in coordinating in-person service delivery, if desired	Voice call or online messenger chat, followed by assistance in coordinating in-person service delivery, if desired. Also included virtual support group sessions.	Online appointment booking and virtual support to coordinate home delivery	Online reservation system; samples collected at laboratory or ART clinic site; laboratory staff coordinated with case managers; tailored support for clients experiencing barriers accessing viral load testing
Indonesia	In-person	Visits to clients’ homes	In-person support through quarterly individual meetings with clients and support group sessions held at the community organization	In-person orders followed by home delivery or clinic visit to pick up refill	Sign-up at clinic
	Virtual	Voice calls or online messenger chats, followed by assistance in coordinating in-person service delivery, if desired. Support was tailored for clients based on their length of prior ART.	Support groups convened over the phone or internet on platforms such as WhatsApp	Online orders followed by home delivery	Online reservation system; samples collected at ART clinic site; clients share results with case managers during VCM sessions

Abbreviations: ART, antiretroviral therapy; VCM, virtual case management.

In Nepal, services were tailored based on client preferences and needs. Clients with the most pressing needs, such as those who had not yet initiated treatment, were experiencing ART adherence issues, or had unsuppressed viral loads were prioritized for VCM. Case managers were provided educational materials and videos, along with scripted, tailored messages to use during their VCM sessions and follow-up (Table 2). During VCM sessions, case managers gathered case details to better address and understand clients’ specific issues.

In Indonesia, new clients were assessed using a case management intake form, via Google Forms, that identified their length of time on ART for further customized support (Table 2). The case management strategy offered differentiated support for clients 0–6 months on ART, 7–12 months on ART, and >12 months on ART. VCM directly assisted clients who required facility referrals, home-based ART services, financial assistance to access services, or other services.

### Process and Activities Documentation

Both EpiC Nepal and LINKAGES Indonesia documented the processes and costs for conversion from in-person to VCM in an analogous fashion. However, the individual activities that supported the transition to VCM differed between the two programs according to existing systems in those locations.

#### Clinical Outcomes Tracking

Client and case manager interactions, including services received and test results, were documented on FHI 360 program tools adapted for online service delivery. In Indonesia, test results for viral load suppression (VLS) and viral load coverage (VLC), which is assessed by the proportion of patients with at least one routine VL test performed within the period of interest, were reported by clients to case managers who then updated the system with results. In Nepal, case managers would receive, log, and report VLS and VLC results to clients, except for clients that tested at the National Public Health Laboratory where results could be shared online directly with the client.

#### Program Cost Estimation

Once all the resource types and quantities were estimated across all activities, a unit cost was assigned to each resource. We estimated activity costs by multiplying the unit cost of a resource by the quantity of the resource required and then summing across all the resources used for an activity. We estimated the cost of setup activities or the cost of operating the online service by summing the costs across the setup activities or the ongoing operation activities. For operating costs, we focused on the average monthly cost for running VCM. The case managers logged all their virtual interactions with clients, and we calculated the cost per PLHIV client receiving VCM. For both startup and ongoing operations, the main resource needed was human resources, which largely already existed. In Nepal, no new case managers were recruited and Indonesia only recruited 14 additional case managers. Both programs also had existing devices (e.g., laptops, tablets, and/or mobile phones) and internet/Wi-Fi packages. The new resources and associated costs were for VCM training, mobile data packages, and SMS fees. Costs associated with other activities, particularly those focused on index testing, home-based ART services, financial assistance for health service utilization, and contact tracing of those who experience treatment interruption through facility partnerships are not included in these estimates.

#### Case Manager Experience

In late 2021, a sample of Indonesian and Nepali case managers providing VCM participated in focus groups and structured interviews to provide feedback on the VCM experience. In Nepal, the case managers were randomly selected, whereas in Indonesia, the CSOS nominated case managers who expressed interest to participate. There was one focus group and 7 interviews in Nepal and 15 interviews in Indonesia. Specifically, 13 case managers serving 7 different districts in Nepal and 15 case managers serving 5 districts in Jakarta participated. They were asked a structured set of open-ended questions about how the transition to VCM affected their work, including what they saw as the biggest challenges and benefits of VCM for their clients and themselves as well as advice about how VCM should be offered to clients going forward.

## Implementation Results and Clinical Outcomes

### Feasibility

During the first 6 months of the pandemic, both programs were able to rapidly transition HIV clients from in-person to VCM. By the end of September 2020, more than 10,000 and 3,000 PLHIV enrolled in the ART centers in project districts in Nepal and Indonesia, respectively, were supported through VCM (Table 3). In both programs, continuity of care remained high, as evidenced by only 1% to 2% interruptions in treatment (IIT). Notably, between October 2020 and March 2021, as COVID-19 restrictions eased in Nepal, fewer clients opted for VCM (86% reduction). During that same period in Indonesia, VCM continued to expand, adding 14 case managers and over 800 new PLHIV clients.

**TABLE 3.** VCM Staff Size and Clients Served During Initial COVID-19 Lockdown (Period 1) and After Restrictions Eased (Period 2), Nepal and Indonesia

**Country**	**Period of Performance**	**Total No. of Case Management Staff**	**No. (%) of Case Managers Providing VCM**	**Total No. of PLHIV on ART**	**No. (%) of PLHIV Receiving VCM Support**	**% IIT Among VCM Clients**	**No. (%) Clients Receiving Home-Delivered ART**	**Case Load**
Nepal	Period 1: Mar–Sep 2020 (6 months)	113 case managers	83 (73.0)	13,391	10,995 (82.1%)	1%	2,836 (21.2)	1:132
	Period 2: Oct 2020–Mar 2021 (6 months)	113 case managers	22 (19.5)	16,679	1,551 (9.3%)	1%	1,475 (8.8)	1:67
Indonesia	Period 1: Mar–Sep 2020 (6 months)	53 case managers	53 (100.0)	7,371	3,801 (51.5%)	2%	6,046 (31.7)^a^	1:72
	Period 2: Oct 2020–Mar 2021 (6 months)	67 case managers	67 (100.0)	7,822	4,656 (59.5%)	1%		1:68

Abbreviations: ART, antiretroviral therapy; IIT, interruption in treatment; PLHIV, people living with HIV; VCM, virtual case management.

a For the period March 2020 through March 2021.

### Program Costs

VCM startup costs under EpiC Nepal and LINKAGES Indonesia were low (i.e., under $2,500) (Table 4). Startup costs were largely related to the development and dissemination of tracking tools and guidance documents. Subsequent ongoing monthly operating costs largely went to support implementing partner staff; this included transportation allowances for staff to provide navigation support to clients (92% of costs). During the first reporting period post-conversion (i.e., March to September 2020), the monthly cost per person to provide VCM was $2.34 in Nepal and $4.67 in Indonesia (Table 4).

**TABLE 4.** Program Startup, Operating, and Unit Costs (US$)

**Country**	**Period of Performance of Service Delivery**	**Startup Costs**	**Average Monthly Operating Costs**	**Average Monthly Cost per PLHIV**
Nepal	March 2020 (1 month)	$1,098		
	Period 1: Mar–Sep 2020 (6 months)		$25,747	$2.34
	Period 2: Oct 2020–Mar 2021 (6 months)		$11,934	$7.66
Indonesia	March 2020 (1 month)	$2,472		
	Period 1: Mar–Sep 2020 (6 months)		$17,711	$4.67
	Period 2: Oct 2020–Mar 2021 (6 months)		$17,091	$3.67

Abbreviation: PLHIV, people living with HIV.

Between October 2020 and March 2021, fewer clients opted for VCM in Nepal, which resulted in an increase to $7.66 in the monthly cost per person supported. During the same period, in Indonesia, there were some efficiency gains as VCM expanded, and the cost per PLHIV client supported per month declined by 20% to $3.67.

### Clinical Outcomes

In both Nepal and Indonesia, rates of VLS reported among the VCM clients indicate that the VCM case managers were able to support their PLHIV clients virtually to maintain adherence and viral suppression (Table 5). Across 3 reporting periods between March 2020 and March 2021, the percentage of VLS was greater than 91%. VLC, however, was low at the onset of the pandemic and increased in subsequent reporting periods, particularly in Indonesia, as testing facilities reopened.

**TABLE 5.** Viral Load Suppression and Viral Load Coverage Among PLHIV Receiving VCM, Nepal and Indonesia

**Country**	**Measurement Month**	**% VLS**	**% VLC**
Nepal	March 2020	95%	10%
	September 2020	94%	Data not available
	March 2021	94%	Data not available
Indonesia	March 2020	91%	60%
	September 2020	92%	60%
	March 2021	92%	62%

Abbreviations: PLHIV, people living with HIV; VCM, virtual case management; VLC, viral load coverage; VLS, viral load suppression.

### Case Manager Feedback

Case managers noted examples of benefits, challenges, and advice regarding the effect of VCM on PLHIV clients and on their own work. In both programs, most case manager responses centered around 4 central issues related to how VCM affected 1) their work conditions, 2) scheduling of and time spent with clients, 3) ability to reach existing and new clients, and 4) sharing of confidential and private information. Several case managers noted that their remuneration may need to be adjusted for provision of VCM. This concern may stem from the fact that case managers are typically compensated for their travel costs and time to reach clients for in-person services, but case managers may receive less of this additional compensation with VCM.

There appeared to be consensus that VCM afforded greater reach to more remote and hard-to-reach clients, allowing the program to reach new clients or clients unreached by traditional approaches. However, some case managers raised the concern that VCM was not as useful or viable with clients who lacked access to mobile phones and/or lacked experience using apps and online platforms. Notably, there was no consensus from the case managers about whether VCM took more or less time to provide than in-person case management nor whether VCM sessions afforded clients more or less confidentiality and privacy to open up about sensitive issues than in-person sessions. Some case managers believed that the virtual consultations gave their clients a greater sense of anonymity and confidentiality enabling them to more freely express their feelings and health concerns, especially when their cameras were turned off. However, other case managers noted that some of their clients were more hesitant to talk openly during virtual consultations, and the case managers felt that face-to-face interaction would have helped to establish trust and security necessary for clients to open up more during sessions.

## Lessons Learned

The transition to telehealth and virtual support services for HIV care during the COVID-19 pandemic is well-documented in high-income settings like the United States. Various studies have reported positive attitudes toward HIV telehealth and positive clinical outcomes (e.g., viral suppression and care retention). However, some studies reported increased loss to follow-up during the COVID-19 pandemic. The United States’ experience highlights the importance of enabling telehealth policies and regulatory environment for sustained virtual services.^25^

The case studies of the EpiC Nepal and LINKAGES Indonesia programs’ transitions to HIV VCM during the COVID-19 pandemic demonstrate that VCM is a feasible and affordable approach to providing support to PLHIV in LMICs. In both programs, HIV case managers were able to quickly transition case management services from in-person to virtual support with low startup costs and small per person monthly ongoing costs. During lockdowns, case managers were able to support large cohorts of PLHIV in routine care and maintain their adherence and viral suppression rates without in-person contact. Given the feasibility and low costs involved in these case studies, we believe the process of VCM rollout documented here should be easily replicable in similar contexts.

These case studies also demonstrate the viability of VCM even in contexts with low or inconsistent internet connectivity because virtual support can still be offered via telephone calls and SMS accessed with a basic feature phone. However, client device ownership and data/Wi-Fi access can influence the type of virtual support that can be offered. Case managers noted the ability of VCM to maintain contact with existing clients and reach those residing further away from service sites or contact new clients. Case managers did, however, note that internet connectivity and mobile phone ownership were not available to all their clients and suggested that both modalities of in-person and virtual be offered to fit different clients’ needs.

Additional challenges observed in the process documentation and noted by the case managers include potential changes to case managers’ compensation structures, digital literacy, and low rates of viral load testing. The latter, however, is inextricably linked with the pandemic itself. Viral load testing was quite low among VCM clients during the first reporting period of the pandemic, particularly in Nepal (10%). These rates of VLC were unavoidable given the closure of HIV testing sites and laboratories, incomplete reporting, and a focus on maintaining PLHIV in treatment rather than viral load tests during this period. As restrictions eased, VLC rates similarly increased.

There are also limitations to the analyses and conclusions that can be drawn from these case studies. During pandemic lockdowns, all in-person case management was suspended precluding a comparison group or cost-effectiveness analysis. Furthermore, to the extent that costs are not captured in an analogous fashion for in-person case management services, a retrospective cost-effectiveness analysis was also not possible. The insights shared from the case managers were not collected in a systematic fashion such that we cannot claim generalizability or preclude the possibility of biases in the sample selected. Also, we were unable to similarly survey clinicians or clients who likely would have provided insights into additional challenges and benefits.

We also recognize that the transition to VCM for EpiC Nepal and LINKAGES Indonesia was facilitated by several unique facets of these programs. In both Nepal and Indonesia, case managers already had and were facile with using mobile devices. The transition to VCM was advanced by demand creation activities. For example, EpiC Nepal identified and mobilized several community members living with HIV who had large social media followings to act as peer champions to promote and influence others to use the project’s VCM services. Also, in both locations, there was an existing online reservation and case management app that could easily be modified to assist with online service reservations and cohort tracking among PLHIV. Specifically, in Nepal, the EpiC project prior to the pandemic had developed an online system for making reservations for HIV testing called MeroSathi, which in Nepali translates to “my friend.” In Indonesia, LINKAGES Indonesia collaborated with the Provincial Health Office in Jakarta to create Jak Support and Jak-Anter. The former is a recovery and resilience emergency fund to help populations at increased risk of HIV to finance care and treatment during the pandemic and the latter, a home-based ART delivery system providing safe and secure delivery of antiretrovirals from facility to client via ride-based apps or transport courier services.

We note that since January 2021, both programs have made some additional modifications that affect the provision of VCM. In Nepal, MeroSathi was upgraded to include case management and tracking functions adapted to more systematically track and support PLHIV, as well as an enhanced risk assessment for new clients. As COVID-19 social restrictions were lifted throughout 2022 and 2023, many case management services transitioned back to face-to-face. However, in both Nepal and Indonesia, a hybrid case management model has become the norm with a mix of face-to-face and virtual channels and services per the clients’ needs and preferences. Reminder and follow-up messages lend themselves particularly well to virtual outreach. VCM is also being used for subpopulations or specific clients that are either better digitally connected or are still not being reached by traditional outreach and services.

These modifications address some of the challenges and corresponding advice offered by the case managers. Challenges remain however with lack of access to digital technologies, particularly lack of reliable internet connectivity, and lack of digital literacy. These challenges, however, are not dissimilar to those faced by most of the world’s population during the rapid conversion to use of digital technologies brought about by the pandemic.^26^ Given the rapid global expansion of internet access and digital technologies, more rigorous implementation documentation and research is needed to more precisely report on and identify the clinical, financial, human resource, and service delivery impacts of VCM for PLHIV, particularly in resource and technology-limited settings.

## Conclusions

Digital connectivity is rapidly expanding across the globe and virtual approaches are increasingly used to generate demand and engage clients into care.^27^ In 2023, internet penetration, which is the proportion of internet users to the total population, was 51.6% in Nepal and 77.0% in Indonesia, corresponding to a 17% and 13% increase, respectively, in each country since VCM was first implemented in 2020.^28^^,^^29^ Recognizing the global trend of rapid digitization, the Nepali National HIV Strategic Plan (2016–2021) suggested that electronic health (eHealth) and mobile health (mHealth) had “transformative potential” to improve health care access and health literacy.^30^ Indonesia has also prioritized electronic and telehealth strategies and has its own national strategy.^31^ Utilizing tools from both eHealth and mHealth, VCM is an approach with transformative potential. VCM expands differentiated service options for PLHIV and their case managers by providing a self-care pathway for engaging with services and receiving support remotely.

These case studies highlight how VCM can contribute to a hybrid service delivery model that expands the reach of services, better meets clients’ needs and preferences, and enhances the abilities of programs to adapt to different contexts and stressors (e.g., disease outbreak, security incidents, natural disasters, etc.). Additionally, these case study results demonstrate that the quality of case management services are not disrupted when shifted to virtual channels and that VCM services can be provided efficiently and affordably, providing rationale for scaled and sustained VCM. Addressing some of the challenges identified via these case studies, including policies around provider compensation for virtual services and digital literacy of clients to effectively engage with virtual services, will support the advancement of VCM. Integration of telehealth and virtual services into national policies related to health service delivery and coordination between often fragmented ministerial departments (e.g., disease areas and IT) are also essential for scaling VCM at a national level. Implementation science research focused on structural barriers and facilitators to VCM could help inform scalability. While case study results documented here focused on the impacts of VCM for PLHIV clients and case managers, we believe offering VCM will, in turn, have benefits to the larger health systems and communities in which HIV programs are situated. Offering additional ways to connect to services has potential to conserve resources, reach new clients, and reduce community transmission.

## Notes

**Peer Reviewed**

## References

[1] Samji H, Cescon A, Hogg RS, et al. Closing the gap: increases in life expectancy among treated HIV-positive individuals in the United States and Canada. PLoS One. 2013;8(12):e81355. 10.1371/journal.pone.0081355. 24367482 PMC3867319

[2] Antiretroviral Therapy Cohort Collaboration; Zwahlen M, Harris R, May M, et al. Mortality of HIV-infected patients starting potent antiretroviral therapy: comparison with the general population in nine industrialized countries. Int J Epidemiol. 2009;38(6):1624–1633. 10.1093/ije/dyp306. 19820106 PMC3119390

[3] Barron M, Mishra V, Lloyd S, Augenstein J. How to measure the value of virtual health care. Harvard Business Review. Published June 24, 2021. https://hbr.org/2021/06/how-to-measure-the-value-of-virtual-health-care

[4] Kay ES, Batey DS, Mugavero MJ. The HIV treatment cascade and care continuum: updates, goals, and recommendations for the future. AIDS Res Ther. 2016;13:35. 10.1186/s12981-016-0120-0. 27826353 PMC5100316

[5] FHI 360. Virtual HIV Interventions: A Budgeting and Programming Aid. Durham: FHI 360; 2022. https://hivpreventioncoalition.unaids.org/en/resources/virtual-hiv-interventions-budgeting-and-programming-aid

[6] Joint United Nations Programme on HIV/AIDS (UNAIDS); World Health Organization (WHO). Virtual Interventions in Response to HIV, Sexually Transmitted Infections and Viral Hepatitis. UNAIDS and WHO; 2022. https://www.unaids.org/sites/default/files/media_asset/policy-brief_virtual-interventions_en.pdf

[7] Willis S, Castel AD, Ahmed T, Olejemeh C, Frison L, Kharfen M. Linkage, Engagement, and Viral Suppression Rates among HIV-Infected Persons Receiving Care at Medical Case Management Programs in Washington, DC. J Acquir Immune Defic Syndr. 2013;64(Suppl 1):S33–S41. 10.1097/QAI.0b013e3182a99b67. 23982662 PMC3844615

[8] Tolley EE, Hamilton EL, Eley N, et al. “The role of case management in HIV treatment adherence: HPTN 078.” AIDS Behav. 2022;26(9):3119–3130. 10.1007/s10461-022-03644-2. 35362913 PMC9371990

[9] Patel P, Kerzner M, Reed JB, Sullivan PS, El-Sadr WM. Public health implications of adapting HIV pre-exposure prophylaxis programs for virtual service delivery in the context of the COVID-19 pandemic: systematic review. JMIR Public Health Surveill. 2022;8(6):e37479. 10.2196/37479. 35486813 PMC9177169

[10] Eisinger RW, Dieffenbach CW, Fauci AS. HIV viral load and transmissibility of HIV infection: undetectable equals untransmittable. JAMA. 2019;321(5):451–452. 10.1001/jama.2018.21167. 30629090

[11] World Health Organization (WHO). Global Strategy on Digital Health 2020-2025. Geneva: WHO; 2021. https://www.who.int/docs/default-source/documents/gs4dhdaa2a9f352b0445bafbc79ca799dce4d.pdf

[12] GSMA. The State of Mobile Internet Connectivity 2021. GSMA; 2021. https://www.gsmaintelligence.com/research/the-state-of-mobile-internet-connectivity-2021

[13] Government of Nepal, Ministry of Health and Population, National Centre for AIDS and STD Control. End Inequalities, End AIDS. Kathmandu: National Center for AIDS & STD Control; 2021. https://www.aidsdatahub.org/sites/default/files/resource/nepal-world-aids-day-2021-factsheet.pdf

[14] Joint United Nations Programme on HIV/AIDS (UNAIDS). HIV AIDS Fact Sheet: Indonesia & Jakarta. UNAIDS Country Office Indonesia; [2021]. https://www.aidsdatahub.org/sites/default/files/resource/indonesia-hiv-aids-factsheets-2021.pdf

[15] Ministry of Health and Population [Nepal], National Centre for AIDS and STD Control. HIV Standard Service Package for Key Populations. National Centre for AIDS and STD Control; 2020.

[16] Ministry of Health, Government of Nepal. National E-Health Strategy 2017. Ministry of Health; 2017. https://dohs.gov.np/content/32/national-e-health-strategy-2017/

[17] Department of Health [Indonesia]. PHO Circular Letter, No. 57 / SE / 2020. Jakarta, Indonesia: Department of Health; 2020.

[18] We Are Social; Hootsuite. Digital 2020: Nepal. Published February 18, 2020. https://datareportal.com/reports/digital-2020-nepal

[19] We Are Social; Hootsuite. Digital 2020: Indonesia. Published February 18, 2020. https://datareportal.com/reports/digital-2020-indonesia

[20] FHI 360, LINKAGES Project. Going Online to Accelerate the Impact of HIV Programs. Washington, DC: FHI 360; 2019. https://nps-info.org/wp-content/uploads/2021/05/resource-linkages-vision-going-online.pdf

[21] FHI 360. Program Data - EpiC Nepal. Nepal; 2020. Unpublished.

[22] FHI 360. Program Data - EpiC Indonesia. Indonesia; 2020. Unpublished.

[23] Coronavirus disease (COVID-19) pandemic. World Health Organization website. Accessed November 24, 2023. https://www.who.int/europe/emergencies/situations/covid-19

[24] Rochmyaningsih D. Indonesia finally reports two coronavirus cases. Scientists worry it has many more. ScienceAdviser. Published March 3, 2020. https://www.science.org/content/article/indonesia-finally-reports-two-coronavirus-cases-scientists-worry-it-has-many-more

[25] Budak JZ, Scott JD, Dhanireddy S, Wood BR. The impact of COVID-19 on HIV Care provided via telemedicine--past, present, and future. Curr HIV/AIDS Rep. 2021;18(2):98–104. 10.1007/s11904-021-00543-4. 33616811 PMC7898490

[26] Gibbs J, Solomon D, Jackson L, Mullick S, Burns F, Shahmanesh M. Measuring and evaluating sexual health in the era of digital health: challenges and opportunities. Sex Health. 2022;19(4):336–345. 10.1071/SH22068. 35970766

[27] Zinck MJ, Minichiello SN, Fick CA, Sawry S, Fonner VA. Virtual case management: a differentiated approach to HIV prevention, treatment, and care. AIDS. 2024;38(2):145. 10.1097/QAD.0000000000003762. 37861692 PMC10734782

[28] We Are Social; Meltwater. Digital 2023: Nepal. Published February 13, 2023. https://datareportal.com/reports/digital-2023-nepal

[29] We Are Social; Meltwater. Digital 2023: Indonesia. Published February 9, 2023. https://datareportal.com/reports/digital-2023-indonesia

[30] Government of Nepal, Ministry of Health, National Centre for AIDS and STD Control. Nepal HIVision 2020: Fast-Track Ending the AIDS Epidemic as a Public Health Threat, by 2030. National HIV Strategic Plan 2016-2021. 2nd ed. Kathmandu, Nepal: National Centre for AIDS and STD Control; 2017. https://reliefweb.int/report/nepal/national-hiv-strategic-plan-2016-2021-nepal-hivision-2020-fast-track-ending-aids

[31] Ministry of Health of the Republic of Indonesia. Blueprint of Digital Health Transformation Strategy 2024. Jakarta, Indonesia: Ministry of Health of the Republic of Indonesia; 2021. https://extranet.who.int/countryplanningcycles/planning-cycle-files/digital-health-transformation-strategy-2024

